# Mathematical modeling for analyzing mass drug administration operational factors for efficient malaria incidence reduction in southern Senegal^☆^

**DOI:** 10.1016/j.idm.2026.01.004

**Published:** 2026-03-02

**Authors:** Khady Ndiaye, El Hadji S. Diop, Hannah C. Slater, Mor A. Loum, Jean-L.A. Ndiaye, Ibrahima Diallo, Medoune NDiop, Standeur N. Kaly

**Affiliations:** a NAGIP-Nonlinear Analysis and Geometric Information Processing Group, Department of Mathematics, University Iba Der Thiam, VCN, BP 941, Thies, Senegal; b PATH, Seattle, USA; c Civil Engineering Department, University Iba Der Thiam, VCN, Thies, Senegal; d Parasitology and Mycology Department, University Iba Der Thiam, VCN, Thies, Senegal; e NMCP, Ministry of Health and Social Action, Dakar, Senegal

**Keywords:** Mathematical modeling, Impulsive differential equations, Mass drug administration, Malaria

## Abstract

Mass drug administration (MDA) has emerged as a promising strategy for reducing malaria incidence in many African countries. A pilot study of MDA in the Tambacounda health district in southeastern Senegal had a significant impact on reducing the malaria burden. This led the Senegalese National Malaria Control Program (NMCP) to consider scaling up MDA to the district level. However, the infrequent use of MDA has resulted in limited knowledge of its use in the specific context of Senegal. In this work, we propose a mathematical model based on impulsive differential equations that integrates asymptomatic carriers to analyze the impact of MDA on malaria incidence in the region. To this end, we explore four different scenarios of MDA operational factors: 1) the number of MDA rounds, 2) the time interval between rounds, 3) the campaign coverage, and 4) the campaign start date. We find that MDA operational factors should not be oversimplified, as they play a critical role in the effectiveness of MDA campaigns. Our model predicts that successful MDA campaigns are obtained with high MDA coverage, multiple rounds within a year, an interval of five to six weeks between rounds, and an intervention that starts in the first month of the transmission period (mid-to-late July). In addition, we find that the starting campaign date and the interval between are highly correlated. These findings suggest that outcomes of MDA campaigns can be improved by optimizing operational factors for specific malaria-endemic settings. When implementing multiple MDA rounds, it is crucial to optimize these factors so that the intervention covers the entire period of high transmission and reaches most of the target population.

## 1 Introduction

Malaria, a disease caused by parasites of the genus *Plasmodium* and transmitted by mosquitoes of the genus *Anopheles*, is one of the world's major public health problems (van der Watt et al., 2022). It remains a leading cause of morbidity and mortality in many tropical and subtropical African regions (Team, 2022a), including Senegal, which accounted for 0.7% of global malaria deaths from 2020 to 2021 (Team, 2022a, 2022b). Despite efforts by the National Malaria Control Program (NMCP) to control the spread of the disease through measures such as the use of insecticide-treated nets (ITNs), indoor residual spraying (IRS), drug-based prevention (intermittent preventive treatment in pregnancy (IPTp), seasonal malaria chemoprevention (SMC)), and case management, southern Senegal remains particularly vulnerable to highly seasonal and heterogeneous malaria transmission. This puts the public health and the well-being of local communities at risk (Legendre et al., 2024).

Since 2000, significant progress has been made in reducing the malaria burden. This has prompted the National Malaria Control Program (NMCP) to establish elimination targets by 2030 (Ciré et al., 2024; National Malaria Control Programme & Ministry of Health and Social Action, 2020; Team, 2024). However, progress toward elimination is slowing down. In this context, mass drug administration (MDA) with dihydroartemisinin-piperaquine (DHA-PQ) and a single low dose of primaquine emerged as a promising intervention to complement existing strategies. MDA aims to reduce the human reservoir of malaria parasites, including asymptomatic carriers, thereby reducing malaria incidence in southern Senegal (Legendre et al., 2024). MDA involves administering a full course of antimalarial medication, such as dihydroartemisinin-piperaquine (DHA-PQ) with the addition of a gametocytocide such as primaquine, to a specific population residing in regions with moderate to high *Plasmodium falciparum* malaria transmission. This is done at regular intervals and around the same time to reduce the disease burden in the short term (Deng et al., 2023). MDA involves systematically distributing antimalarial drugs to a defined population (except for those for whom the drug is contraindicated), regardless of infection status. The goal is to break the parasite's transmission chain (Deng et al., 2023; Gao et al., 2017). Among the artemisinin-based combination therapies (ACTs) used for MDA, DHA-PQ has received particular attention due to its ability to provide long-lasting protection against reinfection and greater post-treatment prophylaxis (Ba et al., 2025; Gerardin et al., 2015; Okell et al., 2014; Zani et al., 1996). The World Health Organization (WHO) has recommended the addition of a single dose of primaquine to an ACT regimen as a gametocytocide against *P. falciparum* infections. However, several studies quantifying the impact of primaquine on ACTs showed that the addition of primaquine did not improve the elimination of parasitaemia that much (El-Sayed et al., 2007) and had minimal effects on the prevalence (Gerardin et al., 2015).

However, the success of MDA campaigns depends on several factors, including coverage of the target population, implementation logistics, and long-term sustainability. Many works have assessed the effectiveness of MDA campaigns in various settings, showing evidence depending on transmission intensity (Gerardin et al., 2015; Okell et al., 2011; Sallah et al., 2018), coverage levels (Brady et al., 2017), programmatic implementation details (Gao et al., 2020), drugs regimen (Dabira et al., 2022; Gerardin et al., 2015; Okell et al., 2014), campaign sustainability and the emergence of resistance (Gao et al., 2020; Maude et al., 2012; Smith et al., 2019). In particular, some interesting works (Brady et al., 2017; Gao et al., 2020; Gerardin et al., 2015) have highlighted the operational challenges of scaling up MDA campaigns. Nevertheless, most of these models are context-specific and do not sufficiently address in details operational challenges, especially in southern Senegal, where MDA with DHA-PQ and a single low dose of primaquine have recently been added to the malaria control strategy intervention package. Questions remain about the optimal conditions for deploying such an intervention in this context. Therefore, it is crucial to understand how to best deploy MDA campaigns for maximum reduction in transmission. These limitations highlight the necessity of modeling frameworks capable of capturing the discrete and time-dependent characteristics of MDA interventions and evaluating their optimal deployment conditions.

Mathematical modeling has long been recognized as a powerful tool for understanding malaria dynamics, predicting the outcomes of interventions, simulating intervention scenarios, and guiding strategic planning (Brady et al., 2017; Djidjou-Demasse et al., 2020; Gerardin et al., 2015). However, only a few mathematical modeling works focused on DHA-PQ-based mass drug administration (MDA) in Senegal, more particularly in the southern regions. A few of these works incorporated impulsive differential equations (IDEs) to reflect the non continuous, time-specific nature of such interventions. For instance, a meta-population mathematical model was used (Sallah et al., 2018) to evaluate chemotherapy interventions, including MDA, targeting stable malaria hotspots. The model was fitted to data from the Mbour health district in central Senegal and A deterministic compartmental model was developed (Slater et al., 2020) to describe the impact of MDA with DHA-PQ and ivermectin on vector survival in a highly seasonal, moderate transmission setting using Data from the Fatick region of central Senegal. Our proposed work differs from these studies in many ways. First, we do not use the same modeling framework or the same drug combination (DHA-PQ plus a single low dose of primaquine). Second, these studies were not conducted in areas with transmission settings comparable to our study area.

Epidemiological compartmental models based on ordinary differential equations are commonly used to model malaria dynamics with applied control measures. However, these approaches often assume continuous dynamics, which do not always reflect the reality of certain public health measures, such as mass treatment or vaccination. In contrast, IDEs-based approaches help model non-continuous phenomena, such as spontaneous changes in the population of infected or susceptible individuals, and optimize the timing and intensity of interventions. This makes it easier to measure the impact of interventions. MDA is a one-time event that occurs at a specific time of year. It results in a sudden reduction in malaria cases after each round. This discrete, seasonal behavior makes IDEs particularly well-suited for capturing malaria dynamics in the presence or absence of MDA implementation and quantifying the impact of MDA campaigns. Several studies have demonstrated the effectiveness of impulsive models in infectious disease contexts. In particular, a susceptible-infected-removed (SIR) hybrid model with impulse vaccination control was proposed (Abouelkheir et al., 2019). Additionally, IDEs were used (Miron & Smith, 2014) to examine the effects of different levels of protease inhibitors in a *T* cell in a mathematical model describing HIV virus dynamics with impulse drug effects. A delayed SEIRS epidemic model with impulse vaccination and varying total population size was proposed (Gao et al., 2007). The obtained results were improved upon in another study (Bai, 2015). Some recent works use impulsive approaches to assess the impact of mass drug administration (MDA) on lymphatic filariasis disease using clinical trial data for drug efficacy (Abdul Halim et al., 2022; Ghosh et al., 2025), and to examine how strategically timed, short-term interventions can optimize visceral leishmaniasis disease control (Awoke et al., 2025). Most of the available malaria-specific models with impulse strategies are based on the vector-host relationship. One such model of malaria transmission dynamics was formulated with the periodic release of Wolbachia-infected mosquitoes at specific times (Kaboré et al., 2024), and the effects of impulsive control strategies on the spread of vector-borne diseases were modeled considering a latent period (Sisodiya et al., 2022). A numerical extension of the periodic Ross-Macdonald model (Gao et al., 2014) determined the optimal timing of MDA for malaria during periods when mosquitoes are not prevalent. The application of such models to MDA in malaria remains limited.

Currently, there is a lack of modeling studies that combine impulse modeling to address the operational challenges of MDA deployment with real data from MDA trial campaigns using DHA-PQ, especially in the Senegalese context. Although a recent compartmental of model of a vector-borne disease (lymphatic filariasis) integrate MDA through time-dependent impulsive function and is calibrated to real data (Ghosh et al., 2025), they do not explicitly model asymptomatic infections and primarily focus on the number of MDA rounds needed to reach elimination, while assuming homogeneous coverage. Moreover, symptomatic individuals are not distinguished from MDA target population. Similarly, related impulsive vaccination models focus on optimal pulsed control strategies within theoretical epidemic frameworks (Abouelkheir et al., 2019), but do not rely on calibration to real field data. In this work, we develop a DHA-PQ MDA compartmental epidemiological model of malaria based on IDEs. Our approach explicitly incorporates asymptomatic carriers, separates routine treatment of symptomatic cases from MDA delivery, and systematically evaluates multiple operational factors beyong the MDA number of rounds. We fit the model to data from the first MDA trial conducted in the Tambacounda health district, located in southeastern Senegal. In addition, to better capture seasonal transmission patterns, our model incorporates temperature-driven variation in malaria transmission. This study aims to provide guidelines for the most effective and optimal deployment of DHA-PQ MDA in southern Senegal through mathematical modeling. Specifically, we investigate how considering different values of MDA operational factors affects the decrease in malaria cases, exploring various scenarios around the coverage, the number of MDA rounds, the interval between rounds, and the timing levels, and evaluating their impact. We discuss the implications of this study for public health and make recommendations for more targeted interventions. Particularly in the context of the ongoing MDA campaigns conducted by the National Malaria Control Program in the Bakel health district of southeastern Senegal as an extension of the initial MDA trial.

The manuscript is organized as follows. Section 2 presents the proposed mathematical model. Section 3 presents numerical simulations of different scenarios, along with a global sensitivity analysis and parameter estimation. Section 4 provides discussions on the obtained results. The paper ends in Section 5 with concluding remarks and some interesting perspectives of this study.

## 2 Mathematical model

In this section, we propose a mathematical model that describes both the natural dynamics of malaria transmission and the sudden changes observed following the MDA implementation. First, we consider a classical host-vector transmission model involving human hosts and mosquito vectors. To derive the proposed model, we demonstrate how the preceding model can be reformulated without considering vector dynamics explicitly. Finally, we will introduce an impulsive model with temperature-dependent transmission parameter to represent the punctual impact of MDA on disease dynamics.

### 2.1 Human-vector (SEACR-SI) model for malaria transmission

We introduce a malaria transmission model with human and vector hosts that integrates asymptomatic carriers under the following assumptions:

Hypothesis 1 :

1. Congenital malaria is not considered. All newborns are susceptible cases.
2. Neither migration nor visitation is considered.
3. A constant population is considered. Therefore, the birth and death rates are equal.
4. Among humans, there are more asymptomatic carriers than clinical infections. Additionally, disease-induced mortality is only correlated with clinical cases.
5. People who are exposed to malaria (*E*) may develop either asymptomatic malaria (*A*) or symptomatic malaria (*C*).
6. Symptomatic cases (*C*) are treated with artemisinin-based combination therapies (ACTs), which are not 100% effective*.* Once cured, they join the recovered compartment (*R*).
7. Asymptomatic individuals (*A*) may be cured naturally. They may also enter the recovered class.
8. For biological considerations, all parameters are positive. The disease-induced mortality rate is the only exception. It can be negative.

Currently, no interventions are considered. The proposed model builds on the recent approach (Aguilar & Gutierrez, 2020).

In the human host, the total human population at time *t*, denoted *N*_*H*_(*t*), is divided into subpopulations of susceptible individuals *S*(*t*), exposed individual *E*(*t*), asymptomatic carriers *A*(*t*), infected individuals with clinical symptoms *C*(*t*), and recovered individuals *R*(*t*). The expression at time t∈R is given by:

(1) $$N_{H}\left(t\right)=S\left(t\right)+E\left(t\right)+A\left(t\right)+C\left(t\right)+R\left(t\right).$$

In the vector host, the total population of mosquitoes at time *t*, denoted *N*_*V*_(*t*), is divided into two subgroups, the susceptible subgroup *S*_*V*_(*t*) and the infected *I*_*V*_(*t*) subgroup. At time t∈R, we have:

(2) $$N_{V}\left(t\right)=S_{V}\left(t\right)+I_{V}\left(t\right).$$

Under Assumption 1 in Hypothesis 1, the subpopulation of susceptible individuals is generated by the number of newborns at time *t* at rate *α*. This population increases due to waning immunity among the recovered individuals at a rate *r*. It decreases due to natural death at rate *m* and due to infection following the contact with infected mosquitoes at rate *β*(*t*), which depends on time to reflect seasonality. Thus, the force of infection in humans, denoted *G*(*t*), is given by:

$$G\left(t\right)=\frac{\beta \left(t\right)\times I_{V}\left(t\right)}{N_{H}\left(t\right)}.$$

Equivalently, we have:

$$G\left(t\right)=\frac{\beta \left(t\right)N_{V}\left(t\right)}{N_{H}\left(t\right)}\times \frac{I_{V}\left(t\right)}{N_{V}\left(t\right)},$$

where for all *t*, we have *β*(*t*) = *ζb*(*t*), with *ζ* representing the proportion of infectious bites and *b*(*t*) being the man-biting rate of mosquitoes. The man-biting rate was assumed to vary over time (in days) to reflect seasonal and environmental influences on the activity of mosquitoes and their host-seeking behavior (Bigoga et al., 2012).

The force of infection is defined as the product of the number of bites per person per unit of time, that is $\frac{b\left(t\right)N_{V}\left(t\right)}{N_{H}\left(t\right)}$, and the proportion of the total number of bites that are successfully infectious for humans given as $\zeta \frac{I_{V}\left(t\right)}{N_{V}\left(t\right)}$.

Let us denote *β*_*h*_(*t*) a scaling factor of transmissibility. We have: $\beta_{h}\left(t\right)=\frac{\beta \left(t\right)N_{V}\left(t\right)}{N_{H}\left(t\right)}$. Then, the expression of *G*(*t*) can be rewritten as follows:

(3) $$G\left(t\right)=\beta_{h}\left(t\right)\frac{I_{V}\left(t\right)}{N_{V}\left(t\right)}.$$

Regarding the dynamics of the vector, the subgroup of susceptible mosquitoes *S*_*V*_(*t*) decreases due to infection following the contact with asymptomatic carriers (*A*) or infected individuals with symptoms (*C*). In addition, the way the disease is transmitted differs between asymptomatic and symptomatic individuals (Assumption 4 in Hyphothesis 1). Thus, the force of infection in mosquitoes is defined as follows:

$$G_{V}\left(t\right)=\beta_{VA}\left(t\right)+\epsilon \beta_{VC}\left(t\right),$$

where *β*_*VA*_(*t*) and *β*_*VC*_(*t*) are the force of infection in mosquitoes from symptomatic individuals (at rate *β*_*vc*_(*t*)) and asymptomatic individuals (at rate *β*_*va*_(*t*)), respectively. *ɛ* is the relative infectiousness of symptomatic infections compared to asymptomatic cases. So that equivalently, we have:

$$G_{V}\left(t\right)=\frac{\beta_{va}\left(t\right)A\left(t\right)+\epsilon \beta_{vc}\left(t\right)C\left(t\right)}{N_{H}\left(t\right)}.$$

Assuming that, without any intervention, all probabilities of mosquito infection are the same, that is *β*_*va*_(*t*) = *β*_*vc*_(*t*) = *β*_*V*_(*t*), *∀ t*; then, the force of infection in mosquitoes is given by:

(4) $$G_{V}\left(t\right)=\frac{\beta_{V}\left(t\right)}{N_{H}\left(t\right)}\left(A\left(t\right)+\epsilon C\left(t\right)\right).$$

The malaria transmission model integrating asymptomatic carriers and involving human and vector hosts is finally defined by the following system of ordinary differential equations:

(5) $$\left\{\begin{array}{l} \frac{dS\left(t\right)}{dt}=\alpha N_{H}\left(t\right)+rR\left(t\right)-G\left(t\right)S\left(t\right)-mS\left(t\right)\\ \frac{dE\left(t\right)}{dt}=G\left(t\right)S\left(t\right)-\delta E\left(t\right)-mE\left(t\right)\\ \frac{dA\left(t\right)}{dt}=p\delta E\left(t\right)-gA\left(t\right)-mA\left(t\right)\\ \frac{dC\left(t\right)}{dt}=\left(1-p\right)\delta E\left(t\right)-\lambda C\left(t\right)-\rho C\left(t\right)-mC\left(t\right)\\ \frac{dR\left(t\right)}{dt}=gA\left(t\right)+\lambda C\left(t\right)-rR\left(t\right)-mR\left(t\right)\\ \frac{dS_{V}\left(t\right)}{dt}=\alpha_{v}N_{V}\left(t\right)-G_{V}\left(t\right)S_{V}\left(t\right)-m_{V}S_{V}\left(t\right)\\ \frac{dI_{V}\left(t\right)}{dt}=G_{V}\left(t\right)S_{V}\left(t\right)-m_{V}I_{V}\left(t\right). \end{array}\right)$$

The first group consists of the fraction of the human host population that is naive and susceptible (S) to infection. Next is the exposed compartment (E), which represents individuals in the liver stage of malaria infection who have not yet developed blood-stage infection. These individuals are infected by the pathogen but are not contagious during the latent period, 1/*λ*. There are also infectious individuals in the blood stage of infection. They may have a symptomatic (class C) or asymptomatic (class A) infection and may have developed simple or severe malaria. By asymptomatic, we mean infected individuals with low parasitemia and no malaria symptoms. Individuals who recover from the infection make up the recovered class (R).

Table 1 describes the parameters of the model 5.

**Table 1:** Dimension of parameters.

Name	Description	Value	Unit	Reference
*α*,*m*	Human natural birth and death rates	1/(67.8∗365)	*day*^−1^	(Web; Web12)
*r*	Waning immunity	1/30	*day*^−1^	expert opinion
*δ*	Incubation rate	1/5	*day*^−1^	(Collins and Jeffery, 1999; Gao et al., 2020)
*p*	Probability of developing asymptomatic infection	0.6	dimensionless	Alonso et al. (2019)
(1 − *p*)	Probability of developing clinical disease: detection probability	0.4	dimensionless	Alonso et al. (2019)
*g*	Recovery rate of asymptomatic individuals	1/160	*day*^−1^	Gao et al. (2020)
*λ*	Recovery rate of infected individuals by medication	1/7	*day*^−1^	Gao et al. (2020)
*ρ*	Disease-induced mortality	6.40/10, 000/365	dimensionless	(Web12)
*ɛ*	Relative infectiousness of symptomatic infections compared to asymptomatic infections	0.9	dimensionless	assumed

The state variables of the model and processes will be described in Section 2.3.2.

### 2.2 Quasi-stationary hypothesis

Modeling of vector-borne diseases, such as malaria, involves populations (humans and mosquitoes) that interact and evolve on different time scales. In such cases, it is possible to introduce two separate temporal scales, allowing one to assume a quasi-stationary behavior for the populations evolving on the faster scale (Brauer, 2019; Brauer & Kribs, 2016; Segel & Slemrod, 1989). A key feature of the model (5) is its different temporal scales (Li et al., 2013), which can be used to analyze its dynamic behavior over time. In the present work, we assume that the mosquito dynamics are quasi-stationary (Li et al., 2013; Tran and Woldegerima, 2025) because the mosquito population evolves more rapidly (approximately four weeks) than the human population (around sixty years). Consequently, the average natural mortality rates of humans and mosquitoes operate on drastically different temporal scales; that is:

$$m\ll m_{V}.$$

Hence, it is reasonable to set:

$$m_{V}=\frac{1}{\phi }m,$$

where 0 < *ϕ* ≪ 1 denotes the ratio of the slow to the fast time scale. We require also that:

(6) $$\beta_{V}\left(t\right)=\frac{1}{\phi }\beta \left(t\right),\;\forall t\geq 0.$$

This scaling assumption naturally induces the following transformation:

$$\alpha =\phi \alpha^{\prime },\;r=\phi r^{\prime },\;g=\phi g^{\prime },\;\lambda =\phi \lambda^{\prime },\delta =\phi \delta^{\prime },\;\rho =\phi \rho^{\prime },$$

and

$$\beta \left(t\right)=\phi \beta^{\prime }\left(t\right),\;\forall t\geq 0.$$

For ease of notation, we still denote *α*′, *r*′, *g*′, *λ*′, *δ*′ and *ρ*′ by *α*, *r*, *g*, *λ*, *δ* and *ρ*, respectively. Thus, system (5) can be written as:

(7) $$\left\{\begin{array}{l} \frac{dS\left(t\right)}{dt}=\phi \left(\alpha N_{H}\left(t\right)+rR\left(t\right)-G\left(t\right)S\left(t\right)-mS\left(t\right)\right)\\ \frac{dE\left(t\right)}{dt}=\phi \left(G\left(t\right)S\left(t\right)-\delta E\left(t\right)-mE\left(t\right)\right)\\ \frac{dA\left(t\right)}{dt}=\phi \left(p\delta E\left(t\right)-gA\left(t\right)-mA\left(t\right)\right)\\ \frac{dC\left(t\right)}{dt}=\phi \left(\left(1-p\right)\delta E\left(t\right)-\lambda C\left(t\right)-\rho C\left(t\right)-mC\left(t\right)\right)\\ \frac{dR\left(t\right)}{dt}=\phi \left(gA\left(t\right)+\lambda C\left(t\right)-rR\left(t\right)-mR\left(t\right)\right)\\ \frac{dS_{V}\left(t\right)}{dt}=\alpha_{v}N_{V}\left(t\right)-G_{V}\left(t\right)S_{V}\left(t\right)-m_{V}S_{V}\left(t\right)\\ \frac{dI_{V}\left(t\right)}{dt}=G_{V}\left(t\right)S_{V}\left(t\right)-m_{V}I_{V}\left(t\right). \end{array}\right)$$

To distinguish between the fast and slow dynamics time scales, let *t* denote the original (fast) time variable, and introduce the slow time variable *ℓ* = *ϕt*. In the following, we relabeled $\beta_{h}\left(\frac{\ell }{\phi }\right)$ and $\beta_{V}\left(\frac{\ell }{\phi }\right)$ to *β*_*h*_(*ℓ*) and *β*_*V*_(*ℓ*), respectively. Thus, in terms of the slow time scale, since for any *X* ∈ {*S*, *E*, *A*, *C*, *R*, *S*_*V*_, *I*_*V*_}, we have:

$$\frac{dX}{dt}=\phi \frac{dX}{d\ell }.$$

Then, system (7) can be expressed as:

(8) $$\left\{\begin{array}{l} \frac{dS}{d\ell }=\alpha N_{H}+rR-G\left(\ell \right)S-mS\\ \frac{dE}{d\ell }=G\left(\ell \right)S-\delta E-mE\\ \frac{dA}{d\ell }=p\delta E-gA-mA\\ \frac{dC}{d\ell }=\left(1-p\right)\delta E-\lambda C-\rho C-mC\\ \frac{dR}{d\ell }=gA+\lambda C-rR-mR\\ \phi \frac{dS_{V}}{d\ell }=\alpha_{v}N_{V}-G_{V}\left(\ell \right)S_{V}-m_{V}S_{V}\\ \phi \frac{dI_{V}}{d\ell }=G_{V}\left(\ell \right)S_{V}-m_{V}I_{V} \end{array}\right)$$

with the initial conditions:

$$S\left(0\right)=S_{0},\;E\left(0\right)=E_{0},\;A\left(0\right)=A_{0},\;C\left(0\right)=C_{0},\;R\left(0\right)=R_{0},\;S_{V}\left(0\right)=S_{0V},\;I_{V}\left(0\right)=I_{0V}.$$

Here, the mosquito population dynamics are treated as quasi-stationary with respect to the slow human time scale. Since the total population of mosquitoes is constant, we can replace *S*_*V*_ by (*N*_0*V*_ − *I*_*V*_), where *N*_0*V*_ represents the fixed total population of mosquitoes. Thus, system (8) can be simplified to:

(9) $$\left\{\begin{array}{l} \frac{dS}{d\ell }=\alpha N_{H}+rR-G\left(\ell \right)S-mS\\ \frac{dE}{d\ell }=G\left(\ell \right)S-\delta E-mE\\ \frac{dA}{d\ell }=p\delta E-gA-mA\\ \frac{dC}{d\ell }=\left(1-p\right)\delta E-\lambda C-\rho C-mC\\ \frac{dR}{d\ell }=gA+\lambda C-rR-mR\\ \phi \frac{dI_{V}}{d\ell }=G_{V}\left(\ell \right)N_{0V}-\left(G_{V}\left(\ell \right)+m_{V}\right)I_{V}, \end{array}\right)$$

with the initial conditions:

$$S\left(0\right)=S_{0},\;E\left(0\right)=E_{0},\;A\left(0\right)=A_{0},\;C\left(0\right)=C_{0},\;R\left(0\right)=R_{0},\;S_{V}\left(0\right)=S_{0V},\;I_{V}\left(0\right)=I_{0V}.$$

Set *x* = (*t*, *S*, *E*, *A*, *C*, *R*) the slow variable and *y* = *I*_*V*_ the fast variable, and define the function $F:R^{6}\times R\to R$ by:

$$F\left(x,y\right)=G_{V}\left(\ell \right)N_{0V}-\left(G_{V}\left(\ell \right)+m_{V}\right)y$$

The critical manifold $M_{0}$ can be taken as:

$$M_{0}≔\left\{{\left(x,y\right)}^{T}\in R^{7}:F\left(x,y\right)=0\right\}.$$

Since $\frac{\partial F\left(x,y\right)}{\partial y}<0$ for all *ℓ* ≥ 0, *A* ≥ 0 and *C* ≥ 0, then, it follows from the theorem of implicit functions that $M_{0}$ can be obtained as:

$$M_{0}≔\left\{{\left(x,y\right)}^{T}\in R^{7}:y=\frac{G_{V}\left(\ell \right)N_{0V}}{G_{V}\left(\ell \right)+m_{V}}\right\},$$

and it is hyperbolically normally asymptotically stable. On the critical manifold $M_{0}$, the slow system becomes:

(10) $$\left\{\begin{array}{l} \frac{dS}{d\ell }=\alpha N_{H}+rR-G\left(\ell \right)S-mS\\ \frac{dE}{d\ell }=G\left(\ell \right)S-\delta E-mE\\ \frac{dA}{d\ell }=p\delta E-gA-mA\\ \frac{dC}{d\ell }=\left(1-p\right)\delta E-\lambda C-\rho C-mC\\ \frac{dR}{d\ell }=gA+\lambda C-rR-mR, \end{array}\right)$$

with

$$I_{V}^{*}\left(\ell \right)=\frac{N_{0v}\beta_{h}\left(\ell \right)\left(C\left(\ell \right)+\epsilon A\left(\ell \right)\right)}{\beta_{h}\left(\ell \right)\left(C\left(\ell \right)+\epsilon A\left(\ell \right)\right)+mN_{H}\left(\ell \right)}$$

and

(11) $$G\left(\ell \right)=\beta_{h}\left(\ell \right)\frac{I_{V}^{*}\left(\ell \right)}{N_{0v}}.$$

From now on, thanks to the obtained results above, we consider a model without mosquito dynamics.

### 2.3 MDA formulation, MDA variables and parameter description

#### 2.3.1 MDA model formulation

Here, we present a mathematical model of malaria dynamics considering MDA delivery via impulsive ordinary differential equations. This modeling approach assumes time-scale separation, wherein the perturbation time scale is small compared to the underlying dynamics time scale. Our goal is to model the natural malaria transmission dynamics on days when there is no MDA delivery (the continuous phenomenon) and sudden perturbations observed in the solutions after a round of MDA delivery with DHA-PQ.

In addition to Hypothesis 1, we also consider the following assumptions to derive our MDA model:

Hypothesis 2 :

1. As in MDA trials, clinical cases (*C*) are not involved in the MDA process.
2. People who receive dihydroartemisinin-piperaquine (DHA-PQ) through the MDA program are considered to be in a prophylactically protected class (*P*_*p*_).
3. The prophylaxis given by the piperaquine (PQ) component of DHA-PQ is gradually lost at a rate *τ*.
4. The rate at which parasites are killed depends on the transmission status of a person (Gao et al., 2020).
5. The addition of primaquine has not been shown to improve the elimination of parasitemia (El-Sayed et al., 2007) and has minimal effects on prevalence (Gerardin et al., 2015). We assume that the addition of primaquine to the MDA regimen has a negligible additional benefit. Therefore, we do not explicitly model its pharmacodynamic effects.
6. Drug action is represented in a simplified manner; curative and prophylactic effects are not modeled separately.
7. For biological considerations, all parameters are assumed to be positive.

We formulate the impulsive malaria model with MDA delivery as a system of IDEs. For further details on IDEs, please see (Lakshmikantham et al., 1989; Stamova et al., 2016).

Let $t\in R^{+}$ and ${\left\{t_{k}\right\}}_{k\in N}$ be the sequence of MDA delivery times. The impulsive MDA model is given by:

(12) $$\left\{\begin{array}{c} \left\{\begin{array}{l} \frac{dS}{dt}=\alpha N_{H}\left(t\right)+\tau P_{p}\left(t\right)+rR\left(t\right)-G\left(t\right)S\left(t\right)-mS\left(t\right)\\ \frac{dE}{dt}=G\left(t\right)S\left(t\right)-\delta E\left(t\right)-mE\left(t\right)\\ \frac{dA}{dt}=p\delta E\left(t\right)-gA\left(t\right)-mA\left(t\right)\\ \frac{dC}{dt}=\left(1-p\right)\delta E\left(t\right)-\left(\lambda +\rho +m\right)C\left(t\right)\\ \frac{dR}{dt}=gA\left(t\right)+\lambda C\left(t\right)-rR\left(t\right)-mR\left(t\right)\\ \frac{dP_{p}}{dt}=-\left(\tau +m\right)P_{p}\left(t\right) \end{array}\right)t\in \left(\left(t_{k},t_{k+1}\right)\right]\\ \left\{\begin{array}{l} S\left(t_{k}^{+}\right)=\left(1-c_{k}\right)S\left(t_{k}\right)\\ \;E\left(t_{k}^{+}\right)=\left(1-q_{E}\times c_{k}\right)E\left(t_{k}\right)\\ A\left(t_{k}^{+}\right)=\left(1-q_{A}\times c_{k}\right)A\left(t_{k}\right)\;\;t=t_{k},\;k=1,2,\ldots ,\\ C\left(t_{k}^{+}\right)=C\left(t_{k}\right)\\ R\left(t_{k}^{+}\right)=\left(1-c_{k}\right)R\left(t_{k}\right)\\ P_{p}\left(t_{k}^{+}\right)=c_{k}\left(S\left(t_{k}\right)+q_{E}E\left(t_{k}\right)+q_{A}A\left(t_{k}\right)+R\left(t\right)\right), \end{array}\right) \end{array}\right)$$

where for any *X* ∈ {*S*, *E*, *A*, *C*, *R*, *P*_*p*_}, $X\left(t_{k}^{+}\right)$ denote the right limit *X* at a time *t*_*k*_. System (12) is supplemented with the following initial conditions:

(13) $$S\left(0\right)=S_{0},\;E\left(0\right)=E_{0},\;A\left(0\right)=A_{0},\;C\left(0\right)=C_{0}\;R\left(0\right)=R_{0},\;P_{p}\left(0\right)=P_{0p}.$$

*c*_*k*_ ∈ [0, 1], *k* = 1, 2, …, denotes the impulsive coverage parameter of the *k*th MDA round.

The total human population is therefore given by:

$$N_{H}\left(t\right)=S\left(t\right)+E\left(t\right)+A\left(t\right)+C\left(t\right)+R\left(t\right)+P_{p}\left(t\right).$$

Finally, the force in humans is defined as in Section 2.2, as:

$$G\left(t\right)=\frac{\beta_{h}^{2}\left(t\right)\left(A\left(t\right)+\epsilon C\left(t\right)\right)}{\beta_{h}\left(t\right)\left(A\left(t\right)+\epsilon C\left(t\right)\right)+mN_{H}\left(t\right)},$$

with the scaling factor of transmissibility given by:

$$\beta_{h}\left(t\right)=\frac{\beta \left(t\right)N_{V}\left(t\right)}{N_{H}\left(t\right)}.$$

*N*_*V*_(*t*) denotes here the total mosquito population at time *t*, which is assumed to be constant, and *β*(*t*) is the transmission rate from vector to human.

Let [0, *t*_max_] be the year interval partitioned into months:

$$0=t_{0}<t_{1}<\cdots <t_{n}=t_{\max}$$

and let $d_{m}=\left(\left[t_{m-1},t_{m}\right)\right)$ denote the days of month *m*, *m* = 1, *…*, *n*.

Let us define the quantity:

$$\tilde{\beta }\left(t\right)=\beta \left(t\right)N_{V}\left(t\right)$$

which we will fit directly from data because *N*_*V*_(*t*) is not known.

Climatic factors such as temperature and rainfall affect significantly the incidence of vector-borne diseases (Agusto et al., 2015). In the case of malaria, temperature is essentially related to mosquitoes and parasite vital rates (Djidjou-Demasse et al., 2020). So, in order to better capture seasonality in transmission, we model $\tilde{\beta }\left(t\right)$ as piecewise constant on each month and driven by the observed average monthly temperature *T*_*m*_ (cf.Fig. 2-(d) in Section 3.2). Several functional forms can be used to model temperature-dependent parameters, such as quadratic, Briere, unimodal, linear, or combination of linear functions (Agusto et al., 2015; Djidjou-Demasse et al., 2020; Mordecai et al., 2013). In our study, we adopt a linear form. Accordingly, we consider a temperature-driven transmission term $\tilde{\beta }\left(t\right)$ of the form:

$$\tilde{\beta }\left(t\right)=\overset{n}{\underset{m=1}{\sum }}\theta_{m}T_{m}1_{d_{m}}\left(t\right),$$

where, *θ*_*m*_ are the month-specific coefficients to be estimated and $1_{d_{m}}\left(t\right)$ denotes the indicator function of the monthly interval *d*_*m*_, taking the value 1 when *t* lies in month *m* and 0 otherwise. more details about the estimation process is given in Section 3.2.

The scaling factor of transmissibility used in this model is then:

$$\beta_{h}\left(t\right)=\frac{\tilde{\beta }\left(t\right)}{N_{H}\left(t\right)}=\overset{n}{\underset{m=1}{\sum }}\frac{\theta_{m}T_{m}}{N_{H}\left(t\right)}1_{d_{m}}\left(t\right).$$

#### 2.3.2 MDA variables and parameters description

In model (12), we investigate the impact of MDA on the dynamics of the epidemic within the human population. So, in addition to compartments define in model 5, we introduce the prophylactically protected compartment (*P*_*p*_), which is the proportion of people under DHA-PQ MDA delivery. They are protected from infection for a period of time before returning to the susceptible class once their protection wears off. The different processes of the MDA model are summarized in Table 2 and all MDA-related parameters are described in Table 3.

**Table 2:** Transitions and rates for the different model states.

Process	Transition	Rate
DHA-PQ MDA delivery (protection gain)	*S* → *P*_*p*_	*c*_*k*_
	*E* → *P*_*p*_	
	*A* → *P*_*p*_	
	*R* → *P*_*p*_	
Waning protection	*P*_*p*_ → *S*	*τ*

**Table 3:** Description of MDA parameters.

MDA Delivery Parameters				
Name	Description	Value	Unit	Reference
*τ*	Daily rate of falling below the minimum effective drug concentration: daily prophylaxis loss	1/30	*day*^−1^	(Gao et al., 2020; Okell et al., 2014)
*q*_*E*_	drug efficacy or probabilities of success of the drug for exposed	95%	dimensionless	(Okell et al., 2011; Sinclair et al., 2009)
*q*_*A*_	drug efficacy or probabilities of success of the drug for Asymptomatics	95%	dimensionless	(Okell et al., 2011; Sinclair et al., 2009)
*N*_0_	Initial set of individuals in the MDA intervention arm villages	10, 045	individuals	trial data
*C*_0_	Initial number of clinical cases	10	individuals	trial data


*c*_1_	Proportion of individuals who received a full dose of MDA round 1	$\frac{6,057}{10,035}$	dimensionless	trial data


*c*_2_	Proportion of individuals who received a full dose of MDA round 2	$\frac{6,836}{10,035}$	dimensionless	trial data


*c*_3_	Proportion of individuals who received a full dose of MDA round 3	$\frac{7,065}{10,035}$	dimensionless	trial data


*c*_*k*_	Mean coverage per MDA round. Proportion of individuals who received a full dose of MDA round *k*	$\left(\left[0,1\right)\right)$	dimensionless	assumed
*Nb*_*R*_	Number of DHAPQ MDA rounds. Each round is a package of three days of directly observed treatment	variable	rounds	assumed
*σ*	Time interval between rounds: number of days between two MDA campaigns	variable	days	assumed
*Start*_*camp*_	Starting campaign date: day of the year when MDA first round is given	variable	days	assumed

**Fig. 1:**
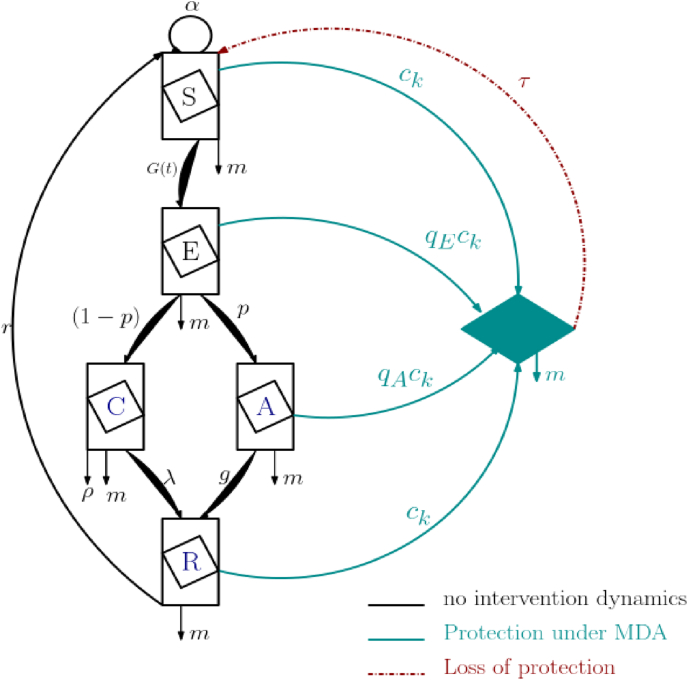
MDA flowchart diagram.

Our model is a semi-continuous system. Without intervention, malaria transmission begins when an infected mosquito bites a susceptible human host. Once the parasites reach liver cells, they progress to the exposed class. From there, they can develop either an asymptomatic infection or clinical disease. Those who develop clinical disease and seek care are treated and join the recovered class. Asymptomatic carriers recover naturally and join the recovered class. However, people in this class become susceptible to infection again due to lack of exposure. Once MDA is delivered, everyone in the human host population receives DHA-PQ, except for those with clinical infections. People from the *S*, *E*, and *A* compartments join the *P*_*p*_ class and become fully protected during the prophylactic period given by the piperaquine component of the drug (30 days on average (Thompson et al., 2022)). They then lose protection progressively. This assumes that the ACT component of the drug successfully clears the infection. For people in the *A* and *E* compartments, we consider a low probability of incomplete parasite clearance by the ACT component of the drug (Gao et al., 2020): $\left(\left(1-q_{A}\right),\left(1-q_{E}\right)\right)$. The dynamics explained above are illustrated in Fig. 1.

**Fig. 2:**
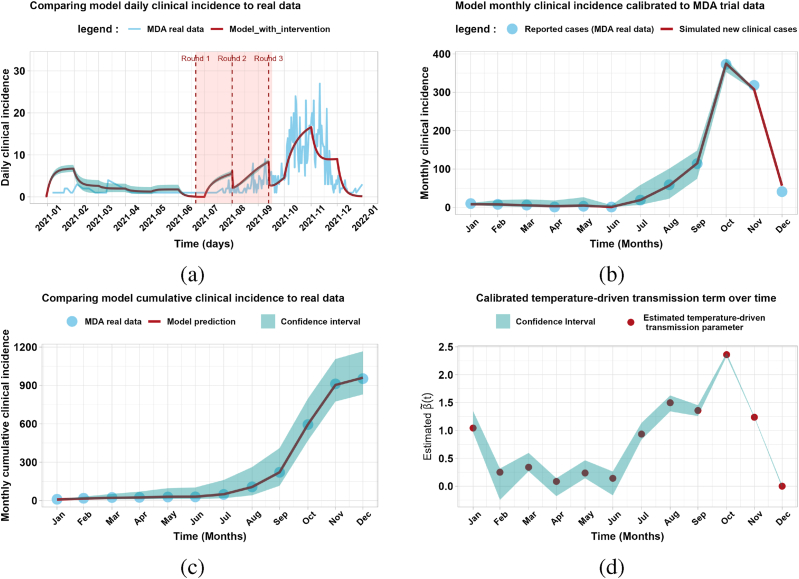
Calibration of the MDA model with real data from the 2021 MDA intervention trial in the Tambacounda health district.

## 3 Numerical experiments

In this section, we present and analyze the results obtained from the numerical simulations of the proposed model. First, we present the data used in the simulations. Secondly, we describe how the model is calibrated and validated. Then, we describe the global sensitivity analysis and discuss the influence of key parameters on model outcomes. To better understand the influence of the parameters on the modeled phenomenon, we explore a wide range of parameter values. The numerical simulations consist of four case studies, or scenarios, in which we focus primarily on optimal parameters directly related to the operational factors of MDA delivery: **1)** the number of rounds, **2)** the time interval between rounds, **3)** the overall campaign coverage, and **4)** the starting campaign date.

### 3.1 Data acquisition

This study used data on daily active malaria cases by age from 30 villages in the Tambacounda health district, located in southeastern Senegal, during the MDA trial study. The data covered three years: 2020 (preintervention), 2021 (intervention), and 2022 (postintervention) (see (Ba et al., 2025) for more information on the study). The daily positive rapid diagnostic tests (RDTs) from the 30 villages in the MDA arm were extracted and aggregated by month to obtain the daily, monthly, and cumulative monthly malaria cases across all villages and age groups. To calculate coverage for each round, we filtered out those who did not consent to receive the drug. Then, we summed up all those who received the drug after each round to calculate the coverage per round.

**Fig. B.11:**
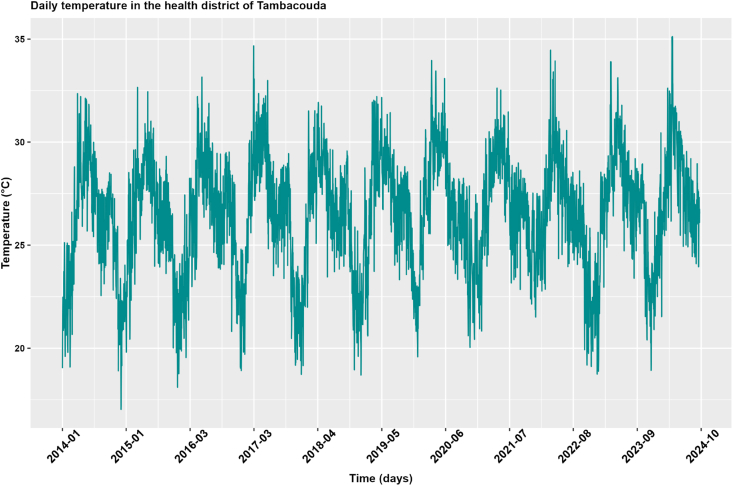
Daily temperature data in the Health district of Tambacounda

Daily temperature data from January 2014 to October 2024 are obtained from Climate Data Store (CDS) - Copernicus (of the European Union & P, 2025). Data were provided in the GRIB format, processed and analyzed using Python, and subsequently converted to CSV format. This study uses the variable *2m temperature* from the downloaded dataset, representing the temperature of the air at 2 m above the surface of land, sea, or in-land waters. Fig. B.11 in Appendix B shows the daily temperature time series for a specific location within the health district of Tambacounda (coordinates: latitude = 13°, longitude = −12°).

### 3.2 Model calibration and validation

To check if the model accurately captures transmission, we run it without intervention for at least three years, until it reaches equilibrium. Then, we calculate the incidence and prevalence starting on the first day of the fourth year. The model yielded an incidence of 336.6 clinical cases per 1, 000 people per year and a prevalence of 22.58%. In the study area, the incidence rate in the pre-intervention year was 202 cases per 1, 000 people per year, and the prevalence rate was 6.2% by microscopy and 22.6% by PCR. Having ensured that the model captures transmission intensity in our study area, we calibrated the model with MDA intervention to the trial study routine data. We used the same MDA parameters for calibration as in the trial study. We simulated three rounds of MDA delivery starting on day 173 (June 23rd of the considered year), with six (6) weeks between rounds.

#### 3.2.1 Estimation of the temperature-driven transmission parameter

Because the vector population *N*_*V*_(*t*) is not directly observed, we estimate the product $\tilde{\beta }\left(t\right)=\beta \left(t\right)N_{V}\left(t\right)$ directly and use it to derive the scaling factor of transmissibility *β*_*h*_(*t*) in the force of infection *G*(*t*). As defined in Section 2.3.1, we model $\tilde{\beta }\left(t\right)$ as piecewise constant on each month and driven by temperature:

$$\tilde{\beta }\left(t\right)=\overset{n}{\underset{m=1}{\sum }}\theta_{m}T_{m}1_{d_{m}}\left(t\right),$$

with unknown month-specific coefficients $\theta ={\left(\theta_{1},\ldots ,\theta_{n}\right)}^{⊤}$ for a given *θ*, the epidemiological model is solved numerically and the cumulative number of new clinical cases predicted at the end of month *m* is denoted *C*_mod_(*m*; *θ*) and given by:

$$C_{\mathrm{mod}}\left(m;\theta \right)=\int_{t_{m-1}}^{t_{m}}\left(1-p\right)\delta E\left(t\right)dt.$$

The coefficients are estimated by minimizing the sum of squared differences between observed monthly cumulative new clinical cases *C*_*obs*_(*m*) and predicted cumulative monthly new clinical cases:

$$J\left(\theta \right)=\overset{n}{\underset{m=1}{\sum }}{\left(C_{\mathrm{mod}}\left(m;\theta \right)-C_{\mathrm{obs}}\left(m\right)\right)}^{2}.$$

$$\theta^{*}=\arg\min_{\theta \in B}J\left(\theta \right),$$

where $B={\left[0,1\right]}^{n}$ imposes admissible constraints. Numerical optimization using the Nelder-Mead optimization algorithm is performed with multiple starting values and convergence diagnostics are reported.

To assess uncertainty in the estimated parameters $\left(\theta_{m}^{*},m\in \left\{1,\ldots ,n\right\}\right)$, we use bootstrap. This approach has been used in many studies (Chowell, 2017; Chowell et al., 2024; Hastie, 2009) to quantify parameter uncertainty and construct confidence intervals in mathematical modeling. It involves generating new data sets by resampling the original data. Then, parameter values are estimated from each of these new bootstrap realizations (Chowell et al., 2024; Hastie, 2009). We generate a bootstrap sample of size *B* = 15, 000 from the best-fit model with an assumed error structure to quantify the uncertainty of the parameter estimates and construct confidence intervals, as explained in (Chowell et al., 2024). Using the best fit, we generate *B* replicated simulated datasets of size *n* (the number of estimated parameters). Next, we refit the model to each bootstrap dataset and examine how the fits behaved over the *B* replications. For each bootstrap sample, we repeat the calibration process. Then, we use the 2.5% and 97.5% (*α*/2 and 1 − *α*/2) percentiles of the re-estimated parameters to construct the 95% prediction intervals. Thus, the confidence interval (CI) is as follows:

$$CI=\left[Q_{\alpha /2},Q_{1-\alpha /2}\right].$$

Fig. 2 shows the MDA model calibrated to real data from the 2021 MDA intervention trial in the Tambacounda health district.

Fig. 2-(a), 2-(b) and 2-(c) compare the daily, monthly and monthly cumulative clinical incidence of the model (red curve) with the daily routine data (sky blue curve). The red lines in Fig. 2-(a), 2-(b) and 2-(c) correspond to the model fits obtained from 15, 000 bootstrap realizations. The green area in all four figures represents the 95% predicted confidence interval. Fig. 2-(d) shows the monthly estimated parameter and the corresponding uncertainty in the estimates.

#### 3.2.2 Goodness of fit

**Fig. 3:**
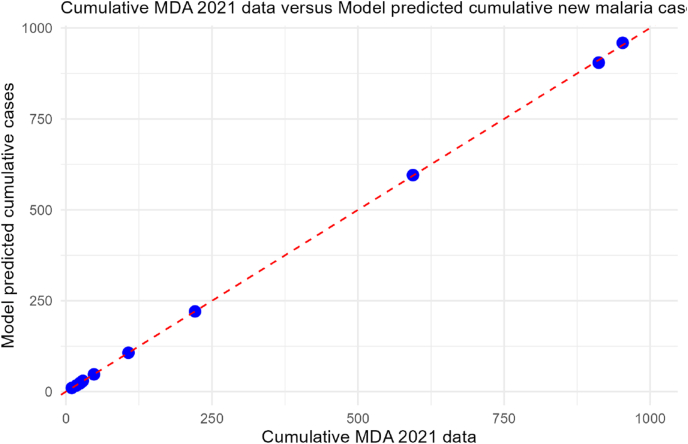
Model goodness-of-fit.

To rigorously assess the validation of the model, we compute the coefficient of determination (*R*^2^) and the root mean squared error (RMSE) between the model-predicted cumulative monthly incidence and the observed MDA intervention data. The cumulative clinical incidence predicted by the model closely matches the observed 2021 MDA data (cf.Fig. 3), with an *R*^2^ equal to 0.99 and an RMSE equal to 2.826. This indicates that 99% of the variability in the observed data is explained by the model. On the other hand, the RMSE provides a quantitative measure of the average deviation. These two goodness-of-fit metrics demonstrate that the calibrated model reliably reproduces the intervention outcomes, providing confidence in the estimated time varying parameter $\tilde{\beta }\left(t\right)$ and, consequently, in *β*_*h*_(*t*), as well as in the model's predictive power.

### 3.3 Global sensitivity analysis

To quantify the influence of key parameters on model outputs, we performed a global sensitivity analysis (GSA). The used approach explores the impact of simultaneous variations of multiple parameters across predefined ranges allowing us to capture nonlinear effects and parameter interactions, which are inherent to complex epidemiological models. Specifically, each parameter was varied within a biologically and epidemiologically plausible range (Table A.4 in Appendix A), and the model was evaluated for all parameter combinations. The two key outcomes were recorded for each simulation. In this work, GSA was performed to quantify the relative importance of model parameters by decomposing the variance of our key outputs, including mainly the annual cumulative new clinical cases and annual prevalence, into contributions from main effects and higher-order interactions. Sensitivity indices were estimated by fitting an analysis of variance (ANOVA), including up to third-order interactions, to the simulation-generated dataset. The fitted ANOVA demonstrated a good goodness of fit, explaining more than 98%(*R*^2^ = 0.984) of the variance of the model outputs. All simulations and analyses were implemented using R software.

The sensitivity analysis focused on two groups of parameters. First, parameters directly related to MDA, including mean coverage per round (*c*_*k*_), starting campaign date (*Start*_*camp*_), number of rounds (*Nb*_*R*_), interval between rounds (*σ*), daily post-MDA loss of prophylaxis (*τ*), and the probabilities of success (*q*_*A*_ and *q*_*E*_), were evaluated for their influence annual cumulative new clinical cases and annual prevalence. Second, parameters governing the generation and dynamics of asymptomatics infections, including parameters *p*, *ɛ*, *g*, *r*, were assessed for their contribution to the same two outcomes.

**Fig. 4:**
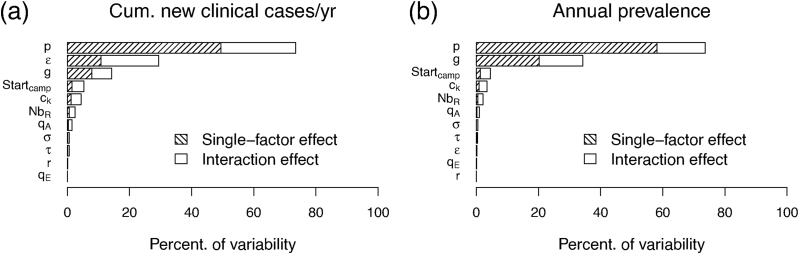
Global sensitivity analysis results.

The GSA shows that the variability of annual cumulative new clinical cases (Fig. 4-a) is largely driven by a small subset of parameters. Specifically, the probability of developing asymptomatic infection *p* explains the largest share of output variance, followed by the relative infectiousness of symptomatic individuals compared to asymptomatic individuals *ɛ* and the recovery rate of asymptomatic individuals *g*. In contrast, parameters directly linked to MDA delivery have a more limited direct effect on cumulative new clinical cases within the explored parameter space. For annual prevalence (Fig. 4-b) the sensitivity differs slightly. *p* remain the main contributor to output variability, followed by *g*, while most other parameters have minor contributions. In addition, interaction effects remain as significant as the single factor effects suggesting that both effects shape the outcomes.

Overall, theses results indicate that while MDA-related parameters influence transmission dynamics, epidemiological parameters governing infection progression (mainly asymptomatic infections) play a dominant role in determining both cumulative incidence and prevalence.

### 3.4 Scenario modeling

In the numerical simulations, we focus on the four main parameters: the number of rounds, the time interval between rounds, the campaign coverage and the starting campaign date. The following four scenarios highlight how these four parameters influence the effectiveness and efficacy of MDA campaigns. As common (Brady et al., 2017), a standard scenario has been used as baseline for comparison. Our baseline is the case of no MDA delivery. In further simulations, we vary the above mentioned operational factors. The outcome metrics of the scenarios are cumulative new clinical malaria cases and the percentage reduction in annual new clinical cases compared to the no-MDA scenario. We will describe each scenario, interpret and discuss the outputs of the simulations before moving to the next one. We start with the number of rounds per year, as the number of chosen MDA campaigns is the most significant determinant of intervention outcomes (Gao et al., 2020).

#### 3.4.1 Scenario 1: number of rounds

**Fig. 5:**
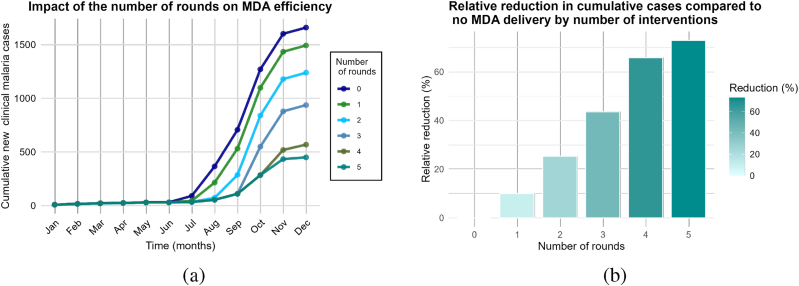
Impact of the number of rounds on MDA campaigns efficiency. (a) Cumulative cases. (b) Relative reduction in annual cases.

In this first scenario, we want to assess the influence of the frequency of MDA campaigns and the number of interventions per year on the reduction of malaria cases. We set the overall coverage to 80%, the interval between rounds to 5 weeks, and the starting campaign date to day 152 of the given year. Then, we chose a range of number of rounds in the set *R* = {0, 1, 2, 3, 4, 5}. For each value in *R*, we plot and compare the cumulative number of new clinical cases given by the model (Fig. 5-(a)). The baseline, or the case of no MDA delivery, serves as the basis for comparison.

The following formula is used to calculate the percentage of reduction compared to no MDA:

(14) $$\text{Relative reduction}=\frac{\left(\text{Annual cases without MDA campaign}\right)-\left(\text{Annual cases for X rounds of MDA}\right)}{\left(\text{Annual cases without MDA campaign}\right)}\times 100$$

where *X* ∈ *R* and *R* = {1, 2, 3, 4, 5} is the set of number of rounds.

Regarding the number of rounds, the scenario shows that the greater the number of rounds, the more efficient the MDA campaign is at reducing clinical malaria cases. One round of MDA results in a reduction of less than 10% compared to no MDA delivery (Fig. 5-(b)).

Therefore, the reduction provided by one round is insignificant. Three rounds with a five-week interval between rounds gives a reduction of less than 50%. Adding just one more round gives a huge difference of more than 60%. However, we notice that there is no significant gain in reduction from four to five rounds. Therefore, when coverage is high (approximately 80%) and a multiple-round MDA campaign with a five-week interval between rounds is implemented, a five-round campaign does not provide much added value compared to a four-round campaign. These results suggest that a one round MDA campaign in the year is not optimal for reducing cases, and adding an extra round to the standard practice of three rounds of MDA can improve the intervention's impact and accelerate elimination. The need to increase the frequency of MDA interventions is justified by the fact that MDA is predicted to be temporary (Brady et al., 2017). In fact, the prophylactic effect of DHAPQ decreases gradually after 30 days (Okell et al., 2014). Additionally, increasing the number of MDA rounds decreases the proportion of the population not reached during the last round (Brady et al., 2017; Gao et al., 2020). Considering that the National Malaria Control Program (NMCP) usually cannot exceed three rounds due to financial constraints (Gerardin et al., 2015), it is interesting to evaluate how the remaining operational factors can be adjusted for optimal impact, as discussed in the following scenarios.

#### 3.4.2 Scenario 2: time interval between rounds

In this second scenario, we will evaluate how the time between two rounds of MDA affects its efficacy. We set the coverage at 80%, with three rounds of MDA per year. Then, we vary the time between rounds from one to eight weeks in one-week increments, for different starting campaign dates between May and August. For each interval, we plot and compare the annual cumulative new clinical cases from the model over time. This process is repeated for each starting campaign date. In this scenario, the case of no MDA delivery was also the baseline.

**Fig. 6:**
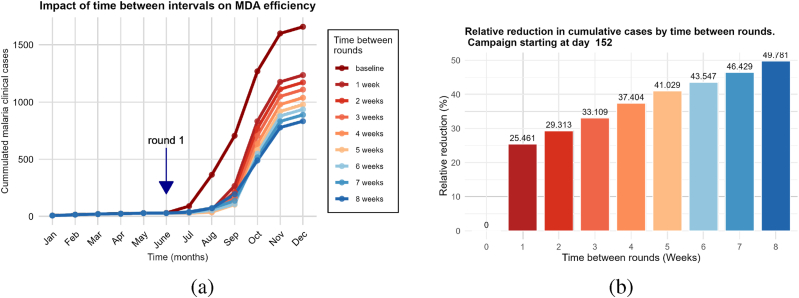
Impact of the time between rounds on MDA campaigns efficiency. (a) Cumulated cases. (b) Relative reduction in annual cases.

The simulations show that starting MDA campaigns earlier, before the transmission period, results in a greater reduction in cases when the rounds are spaced farther apart (Fig. 6-(a)).

**Fig. 7:**
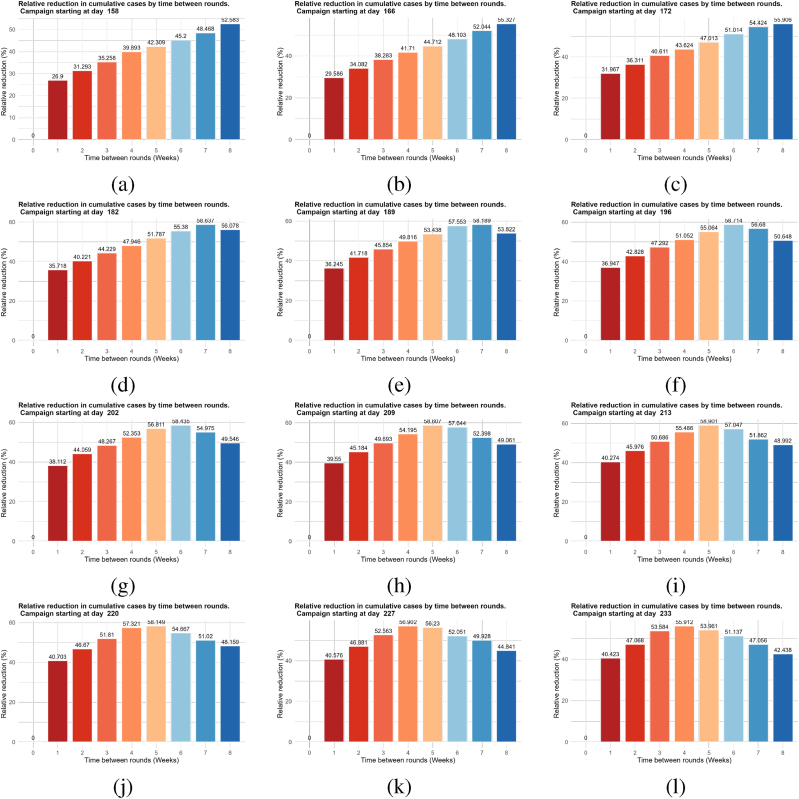
Percentage reduction in annual cumulative new clinical cases compared to no MDA per time between rounds (in weeks) for various campaign start dates.

For three rounds of MDA, when started earlier before the transmission period (between the end of May and the end of June), widely spaced rounds of MDA are more efficient (Fig. 6, Fig. 7). In contrast, when the campaign starts just at the beginning of the transmission period (July) (Fig. 7-(e), 7-(f), 7-(g), and 7-(h)) or a few weeks after the beginning of the transmission period (August) (Fig. 7-(i), 7-(j),7-(k) and 7-(l)), closely spaced rounds of MDA (*i.e.*, four to six weeks) have a more significant effect. Moreover, the simulations show that the optimal reduction is achieved for a three-round MDA when the campaign starts at the end of July with an interval of five to six weeks between rounds (Fig. 7-(i)). This ensures that the period of high transmission (from September to October) is covered by the MDA campaigns. Overall, the main conclusion to retain from this scenario is that, for a three-round MDA campaign, spacing the rounds so that the peak of transmission is covered by the end of the third round is associated with a greater decrease in new clinical cases. Furthermore, this scenario shows that, when considering multiple rounds of MDA per year and high overall coverage, the impact of the time between rounds on MDA efficacy depends heavily on the choice of starting campaign date. The obtained results agree with previous modeling observations (Brady et al., 2017; Gao et al., 2020; Gerardin et al., 2015).

#### 3.4.3 Scenario 3: campaign coverage

**Fig. 8:**
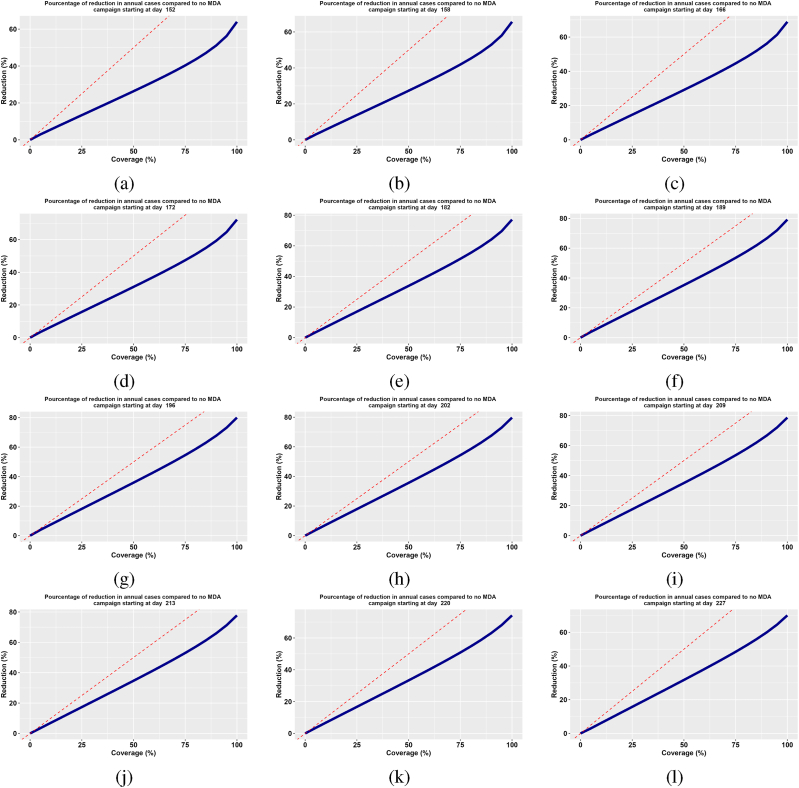
Impact of coverage on the effectiveness of MDA campaigns.

Coverage is defined as the proportion of the population that receives an intervention. It is one of the main determinants of the success of mass drug administration (MDA) campaigns (Awoke et al., 2025; Gao et al., 2020; Gerardin et al., 2015). In this scenario, we assess how coverage affects the success of MDA. We ran a three-round MDA with a five-week interval between rounds. Then, we varied the overall coverage levels from 0 to 100% and computed the percentage reduction in annual cases compared to no MDA using the model outcomes. This process is repeated for different starting campaign dates. Finally, we plot the evolution of the reduction rate against the overall coverage rate. In this scenario, we assume that the proportion of the target population receiving MDA remains consistent across rounds to maintain the same overall coverage. The simulation results illustrated in Fig. 8 reveal that regardless of the starting campaign dates, the greater the coverage, the greater the reduction in annual new clinical cases. There is clearly a correlation between coverage and case reduction. Additionally, depending on the starting campaign date, the percentage reduction achieved at different coverage levels varies. When coverage is very high (up to 100%), reductions of up to 70% are achieved when the campaign starts in June or after the first week of August (Fig. 8-(a), 8-(b), 8-(c), 8-(d), 8-(k) and 8-(l)). In contrast, the reduction in annual new clinical cases is optimal when the MDA campaign starts in July and the first week of August, reaching up to 80% (Fig. 8-(e), 8-(f), 8-(g), 8-(h), 8-(i) and 8-(j)). Therefore, achieving high coverage is essential to ensuring widespread protection, interrupting disease transmission, safeguarding vulnerable groups, and ultimately reducing the overall disease burden (Awoke et al., 2025). Another observation from the results is that even with 100% coverage, the reduction percentage does not reach 100%. This can be explained by the fact that, in the model, we assumed a 5% probability of therapeutic failure of DHA-PQ among exposed and asymptomatic individuals. Additionally, clinical cases are treated directly after symptoms appear.

#### 3.4.4 Scenario 4: starting campaign date

In the context of malaria elimination through MDA, one of the research priorities is defining starting thresholds for MDA. Due to the highly seasonal nature of malaria transmission in southern Senegal, the timing of MDA implementation could greatly impact the intervention's overall effectiveness. In this last scenario, we evaluate how the start date of the campaign affects the intervention's effectiveness. We simulate several options with the MDA campaign beginning in different months of the year to identify the optimal time to reduce clinical incidence. We fix the overall coverage to 80%, the number of rounds to three, and set a range of starting campaign dates depending on the transmission period of the study area: before, earlier, just before, and in the middle of the transmission period, from March to the end of August of the intervention year. We test the scenario with different time intervals between rounds, ranging from three to seven weeks. Then, as in the previous scenarios, we compare the annual cumulative new cases and the percentage of reduction.

**Fig. 9:**
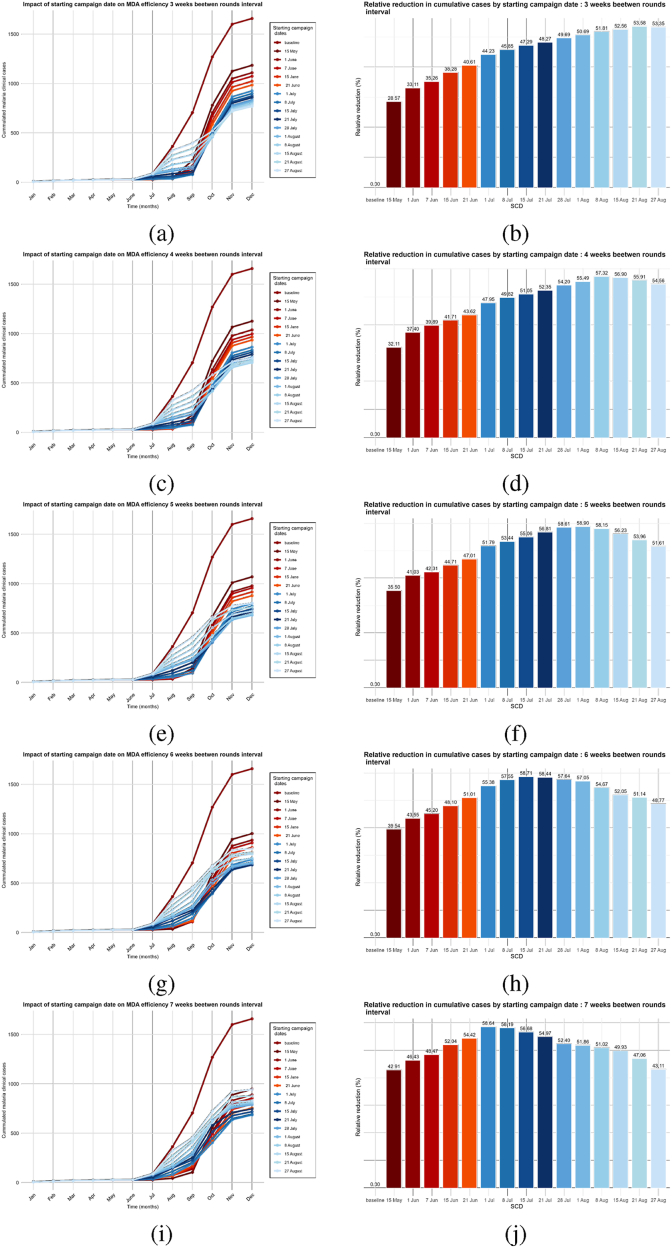
Impact of the starting campaign date on MDA campaigns efficiency.

As can be seen in Fig. 9, the optimal starting campaign date depends heavily on the interval between rounds considered.

**Fig. 10:**
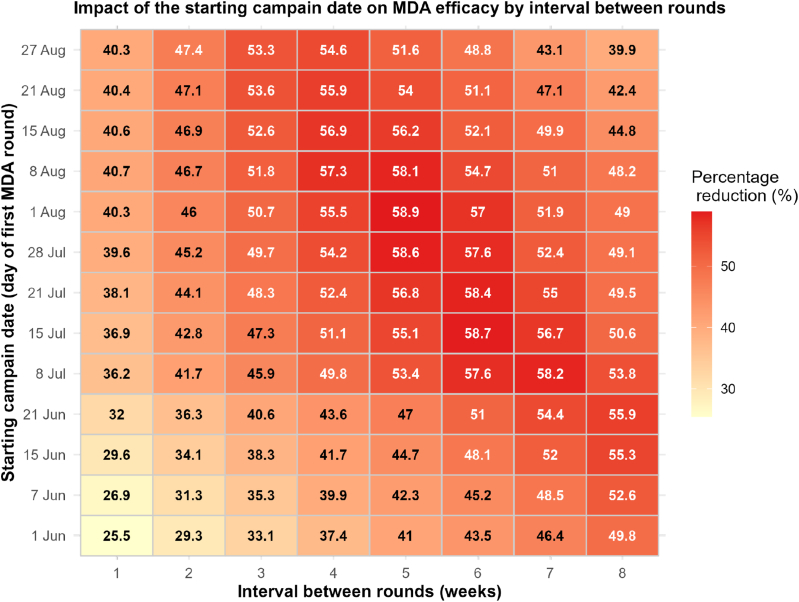
Impact of starting dates and interval between rounds on MDA efficiency.

When a three-week interval between rounds is considered, the proposed model predicts that starting MDA in August results in higher reductions in malaria cases, with the optimal reduction (53%) achieved in the last week of August (Fig. 9-(a) and Fig. 9(b)). If a four-week interval between rounds of MDA is considered, starting between the first and second weeks of August results in a higher reduction of about 57.32% (Fig. 9-(c) and Fig. 9-(d)). If the MDA rounds are spaced five weeks apart, starting the campaign at the end of June reduces 58.9% of new clinical malaria cases (Fig. 9(e) and 9-(f)). Assuming a six-week gap between successive MDA rounds, our model predicts that starting in the second week of June is optimal, achieving a reduction of about 58.71% (Fig. 9(g) and 9-(h)). When a seven-week interval between campaigns is considered, the predicted optimal starting campaign date is in the first week of June, with a maximum reduction of about 58.64% (Fig. 9(i) and 9-(j)). Also, we observe that among all the combinations of campaign date and between rounds tested in this scenario, optimal reductions are obtained when MDA campaigns start during the month of July. Moreover, the best reductions are obtained when MDA campaigns start in the last week of July with 5 or 6 weeks between rounds of MDA (Fig. 10). Furthermore, the greatest reductions are obtained when the MDA campaign starts in the last week of July with five or six weeks between rounds. Fig. 10 summaries the possible obtained reduction depending on the starting campaign dates and the interval between rounds.

## 4 Discussions

We develop and analyze in this work a malaria transmission model that incorporates IDEs. The goal is to evaluate the impact of key operational factors, such as the number of MDA rounds, the interval between rounds, the coverage rate, and the campaign start date, on the effectiveness of MDA campaigns in southern Senegal. Numerical simulations were performed using data from the first MDA pilot study in the region. The outcomes were assessed using the annual cumulative number of new clinical malaria cases and the percentage reduction in incidence compared to the no-MDA baseline.

Our results indicate that increasing the number of MDA rounds significantly reduces clinical malaria cases, especially when the number of rounds exceeds three. However, the marginal gain between four and five rounds is limited. This suggests that four rounds may be the optimal balance between impact and resource constraints. Simulations also show that the time interval between rounds is crucial: for three-round campaigns, an interval of five to six weeks provides the greatest reduction when MDA begins at the end of July. This ensures the period of peak malaria transmission (typically from September to October) is covered by the prophylactic effect of the used drug combination. These findings are consistent with existing modeling works (Brady et al., 2017; Gao et al., 2020; Gerardin et al., 2015), which also emphasized the importance of aligning MDA timing with the transmission seasonality.

The results further highlight the importance of achieving high coverage for MDA success. The reduction in annual clinical cases increases almost linearly with coverage, confirming that incomplete participation can undermine the intervention's overall impact. However, even with 100% coverage, the model does not show complete elimination of cases due to the assumed 5% probability of therapeutic failure and the persistence of asymptomatic infections. These findings underscore the importance of strategies that promote high participation and adherence throughout all MDA rounds.

The start date of the campaign is also crucial. Simulations suggest that launching MDA in mid to late July, with a five-to six-week interval between rounds yields the greatest reduction in malaria incidence. This timing allows the last MDA round to coincide with the onset of peak transmission, thereby maximizing the prophylactic coverage during the critical period. These predictions align with field study observations in comparable transmission settings (Awoke et al., 2025; Brady et al., 2017; Gerardin et al., 2015), reinforcing the reliability of our model's conclusions.

Global sensitivity analysis allowed us to quantify the influence of keys parameters on annual new clinical cases and annual prevalence. The analysis suggested that while high MDA coverage, appropriate timing, and repeated rounds remain necessary, their effectiveness depends critically on the underlying epidemiological context. For instance, in settings characterized by higher transmission potential or slow infection clearance, improvements in MDA delivery may yield limited gains unless complemented by strategies that reduce susceptibility or transmission.

Beyond identifying optimal operational parameters, this study contributes to the understanding of how impulsive interventions, such as MDA, can be effectively modeled within malaria transmission dynamics. IDEs provide a realistic mathematical framework for capturing the discrete, time-limited nature of MDA events, which are not adequately represented in continuous-time models. Compared with recent IDE-based models calibrated to real data (Ghosh et al., 2025), our model explicitly incorporates asymptomatic infections and distinguishes MDA from routine treatment of symptomatic individuals capturing a key programmatic distinction often overlook. While previous interesting studies used an individual-based, spatially explicit framework including logistics, human mobility, and transmission heterogeneity, they did not examine detailed operational factors. Our approach provides a more operationally detailed perspective allowing us to examine how coverage, timing, inter-round intervals, and campaign start date jointly shape MDA effectiveness.

However, several limitations of the current modeling framework must be acknowledged. First, the quasi-stationary hypothesis, which was adopted to simplify mosquito dynamics, assumes that the total mosquito population remains constant over time. While this assumption allows for analytical simplifications and focuses attention on human infection dynamics, it neglects seasonal fluctuations in mosquito abundance, which can substantially influence malaria transmission intensity. As way to partially account for seasonality, we included a temperature-dependent transmission term in the force of infection, reflecting variations in vectorial capacity over time. Second, the model assumes homogeneous coverage across rounds, but in practice, participation may decline over successive rounds. Incorporating variable coverage could improve predictive accuracy. Third, the drug efficacy parameters and the immunity-related transition rates were based on literature estimates rather than field-calibrated data, which introduces uncertainty into the quantitative results. Collecting additional field data are important steps to refine parameter estimates and validate the model's predictions. Fourth, Drug action is represented in a simplified way, with fixed drug efficacy probabilities and constant prophylaxis loss rate, without explicitly modeling pharmacokinetic variability, emergence of drug resistance, or distinct curative and prophylactic effects of DHA-PQ.

Despite its limitations, the model offers valuable insights into designing effective MDA strategies. The results suggest that increasing coverage and aligning the timing and frequency of MDA with the local transmission season can substantially enhance the impact of interventions. From a policy perspective, these findings can inform the National Malaria Control Program (NMCP) as it optimizes the operational planning of MDA campaigns in southern Senegal and other endemic regions.

## 5 Conclusion and perspectives

In this work, we have proposed a mathematical model of malaria transmission to assess how MDA operational factors influence the success of mass drug administration (MDA) campaigns. This study contributes to the overall understanding of malaria transmission in southern Senegal and the optimization of MDA delivery. Using the annual cumulative number of new clinical cases and the percentage reduction in annual new clinical cases compared to no MDA as outcome measures, we have identified the determinants of MDA effectiveness and optimal operational factors in the Senegalese context. Our model has predicted that regardless of the campaign start date, the higher the coverage, the greater the impact of MDA, although the impact is more significant when campaigns start during the transmission period. In addition, the percentage reduction is predicted to be highest when multiple rounds of MDA are implemented closely spaced (within 5 to 6 weeks) throughout the year. Furthermore, the start date of the campaign has a large impact on the effectiveness of MDA. Finally, when multiple rounds of MDA are considered, scaling up campaigns to start in the transmission period and cover the season of maximum malaria transmission may make MDA campaigns more successful. Most of these results are consistent with findings in the literature. Therefore, the results of this mathematical modeling approach will help inform policy decisions by providing insights into the relative effectiveness of MDA. Global sensitivity analysis results emphasize that MDA planning should integrate both operational optimization and context-specific epidemiological characteristics to achieve durable impact. Ongoing work includes incorporating age into the model, performing mathematical analysis of the model, and analyzing the cost-effectiveness of the intervention for the different scenarios proposed. As for perspectives, it would be interesting to evaluate the effect of different coverages for each round of MDA in the case of multiple rounds of MDA to improve the predictions. In addition, to include other existing interventions in the model to assess the combined effect of MDA and other strategies, such as vector control. It may also be useful to assess how long MDA campaigns should be repeated to achieve elimination targets. Furthermore, Future model extensions could incorporate a pharmacokinetic–pharmacodynamic sub-model to explicitly represent the curative and prophylactic effects of DHA-PQ and their decay over time.

## CRediT authorship contribution statement

**Khady Ndiaye:** Writing – original draft, Methodology, Investigation, Formal analysis, Conceptualization. **El Hadji S. Diop:** Writing – review & editing, Visualization, Validation, Supervision, Methodology, Investigation, Formal analysis, Conceptualization. **Hannah C. Slater:** Validation, Conceptualization. **Mor A. Loum:** Software. **Jean-L.A. Ndiaye:** Validation, Funding acquisition, Conceptualization. **Ibrahima Diallo:** Validation, Resources. **Medoune NDiop:** Resources. **Standeur N. Kaly:** Validation, Resources.

## Data availability statement

Data will be made available on reasonable request.

## Funding

This study was funded by a grant from the Gates Foundation (INV-047051). The funders had no role or influence on the design and interpretation of the data collected, as well as in writing the manuscript.

## Declaration of competing interest

The authors declare that they have no known competing financial interests or personal relationships that could have appeared to influence the work reported in this paper.

## Figures and Tables

**Table A.4:** Model parameters and ranges considered in the global sensitivity analysis (GSA).

Parameter	Description	Range
*q*_*E*_	Probabilities of success of the drug for exposed	[0.05, 0.99]
*q*_*A*_	Probabilities of success of the drug for asymptomatic	[0.05, 0.99]
*ɛ*	Relative infectiousness of symptomatic infections compared to asymptomatic cases	[0.05, 1]
*r*	Waning immunity rate	[1/365, 1/15]
*τ*	Rate of loss of post-MDA prophylaxis	[1/50, 1/15]
*σ*	Interval between rounds	[7, 8 × 7]
*p*	Probability of developing asymptomatic infection	[0.05, 0.98]
*g*	Recovery rate of asymptomatic	[1/365, 1/30]
*c*_*k*_	MDA coverage	[0.1, 0.99]
*Nb*_*R*_	Number of MDA rounds	[1, 5]
*Start*_*camp*_	Start day of MDA campaign	[1, 365]
